# Not All Offspring Are Created Equal: Variation in Larval Characteristics in a Serially Spawning Damselfish

**DOI:** 10.1371/journal.pone.0048525

**Published:** 2012-11-14

**Authors:** Jessica Claire Maddams, Mark Ian McCormick

**Affiliations:** School of Marine and Tropical Biology, James Cook University, Townsville, Queensland, Australia; Institute of Marine Research, Norway

## Abstract

The way organisms allocate their resources to growth and reproduction are key attributes differentiating life histories. Many organisms spawn multiple times in a breeding season, but few studies have investigated the impact of serial spawning on reproductive allocation. This study investigated whether resource allocation was influenced by parental characteristics and prior spawning history in a serial spawning tropical damselfish (*Pomacentrus amboinensis*). The offspring attributes of isolated parents of known characteristics were monitored over a 6-week breeding period in the field. Smaller females produced larvae of longer length and larger energy reserves at hatching. This finding is contrary to several other studies that found larger females produce offspring of greater quality. We found that resource allocation in the form of reproductive output was not influenced by the number of spawning events within the breeding season, with larger females producing the greatest number of offspring. Larval characteristics changed as spawning progressed. There was a general decline in length of larvae produced, with an increase in the size of the larval yolk-sac, for all females regardless of size as spawning progressed. This trend was accentuated by the smallest females. This change in larval characteristics may reflect a parental ability to forecast unfavourable conditions as the season progresses or a mechanism to ensure that some will survive no matter what conditions they encounter. This study highlights the importance of accounting for temporal changes in reproductive allocation in studies of reproductive trade-offs and investigations into the importance of parental effects.

## Introduction

A central question in life history theory is how individuals allocate resources between current reproduction and future survival [1], [2]. One of the basic principles of life-history theory is that reproduction carries a cost for subsequent survival and therefore for individual fitness [1], [2]. This cost may lead to a trade-off between reproductive effort at any given time, and the anticipation of future reproduction [3], [4]. Given that reproduction is a costly process where resources have been temporarily sacrificed or traded-off from other life functions [5], [6], energy invested into reproduction should have a fitness benefit.

Reproduction in the simplest sense can be measured by the number of offspring produced, and the characteristics of those resulting offspring. Both of these measures of reproductive allocation have been reported to vary seasonally in a diverse group of organisms (e.g. insects, fish, amphibians and reptiles). In general, variations in offspring number or characteristics have been attributed to genetic or non-genetic parental effects [7]–[10]. Of these effects, maternal influences are regarded as of particular importance due to the females' large investment of energy into eggs [11]. Maternal effects may influence the developmental schedules [12], [13], growth [14]–[16] and performance [17], [18] of offspring, and inevitably the numbers that survive to contribute to the next generation. Maternal investment to reproduction may change in response to her size [12], [19], [20], age [21]–[23] or body condition [24]–[26], and may also be influenced by the environmental conditions she experiences [27]–[34]. Ultimately, her reproductive fitness results from a balance between the cost to herself and the gain to her offspring. While it is known how resource allocations to offspring change with age, size and social status of parents for a diverse array of taxa (e.g. butterflies, sheep mites, fishes) [8], [35], little is known of whether or how allocations change among successive reproductive events within a breeding season.

Early stages (i.e. eggs and larvae) of teleostean fishes are often subject to high mortality [36]–[38], such that very large investments in reproductive material are necessary to ensure the persistence of populations [39]. Producing very high numbers of offspring is a common reproductive strategy among a variety of reef fishes with pelagic eggs or larvae [40], as it is with other organisms with complex life cycles (e.g. many insects and amphibians). Most coral reef fishes spawn more than once over a breeding season. This is a strategy common to other taxa, for example 64 species of Neo-tropical frogs [41] and at least 10 lizard families produce multiple clutches in a breeding season [42]. Spawning in batches over a period of several weeks is considered to be one factor that may actually increase the probability of larval survival by enhancing the chance to encounter optimal feeding conditions [31], [43]. Currently, our understanding of energy allocation to reproduction over successively produced clutches is limited. It has been stated that the difficulty associated with accurately recording fecundity estimates for sequential spawning species has excluded this reproductive method from being appropriately modelled in current life history theory [39].

Evidence suggests that parents have the ability to adjust their resource allocations within a breeding season [43]–[45]. It has been suggested that resource competition between offspring can cause selective variation in offspring characteristics. For example, if resource competition between offspring is high, mothers may increase their per offspring investment as the season progresses to ensure that offspring spawned later in the season will not be at a competitive disadvantage [31], [43]. This indicates that mothers have the ability to recognise prior energy investment to offspring and to alter their offspring accordingly. Alternatively, it has been suggested that variation is a form of risk spreading ensuring that no matter what conditions the offspring encounter some will survive [46]. Whether or not the parents actually make a tactical decision to adjust resource allocation or if the response is purely a product of environmental conditions (and the fish's physiological reaction to these), such as an increase in food supply [26], [47] is difficult to determine.

Without an understanding of how an individual's history of prior spawning, either within or between breeding seasons, has influence on subsequent spawning events it is impossible to realistically parameterise an essential component of life history theory. In an energy limited environment [48], we may predict that investment into egg production in serially spawning organisms may diminish with each successive spawning bout within a breeding episode.

The objective of the present study was to examine the influence of parental characteristics on reproductive output and larval characteristics of a serially spawning marine teleost, the benthic spawning damselfish, *Pomacentrus amboinensis* (Pomacentridae, Bleeker 1868). As such, this is the first field-based study to assess how the energy allocated to reproduction changes throughout a breeding season in a serial spawning marine teleost.

## Methods

### Study species

*Pomacentrus amboinensis* was selected as an example of a serially spawning marine teleost with a complex life cycle for a number of reasons: (1) it has a small home range, is site attached and can be transplanted onto patch reefs made of coral and rubble; (2) the female lays a monolayer of eggs on a nesting surface guarded by the male prior to hatching, so that the total number eggs and their development can be readily quantified; (3) the fertilized eggs hatch after about 5d (at 28°C) at ∼20 min after sunset allowing for easy access to newly hatched larvae; (4) the species is widespread throughout the Indo-Pacific and has life history features that are commonly displayed by many other fishes. Over the breeding season this species can spawn several times. The present study encompassed the majority of the spawning season at the study site. Field observations of spawning before and after the six week study found no evidence of spawning prior to the start of the project, and very little after its completion. This species is protogynous hermaphrodite living in groups typically containing one male, which guards a benthic nest site, and one to seven females [49]. Tagging studies using visible tags and passive integrated transponder tags have found that females do not travel more the 12m along a continuous reef to visit male nest sites [49], [50]. To be able to accurately determine the parentage of the eggs it was necessary to isolate breeding pairs by transplanting them to patch reefs established on a sand-flat. Patch reefs are a part of the habitat they normally occupy [51]. Previous studies that have individually tagged pairs have shown that the fish do not move the 20 m distance between patches or the reef edge [50], [52]. A study on the demography of the species at the study reef found that females become reproductively mature at ∼2 year old, and reach a maximum age of 6 years on the fringing reef around Lizard Island (McCormick unpublished data). Once the larvae hatch from the benthic nests they spend between 18–23 days as pelagic larvae before settling back to a reef habitat [53].

### Study site

The study was conducted on Lizard Island (14°40′449S, 145°27′400E) in the northern Great Barrier Reef, Australia, over a six week period (October to December 2006). A matrix of 40 patch reefs was constructed on sand at a depth of 2–3 m within the blue lagoon. Each patch reef was composed of live and dead coral (*Pocilliopora damicornis*) with a basal area of approximately 2 m^2^ and extended approximately 40 cm above the substrata. Adjacent patch reefs were separated by 20 m of sand and were a minimum of 20 m away from contiguous reef. A 250 mm length of PVC pipe (170 mm diameter) was positioned within each patch reef to provide an artificial nesting surface. The artificial nests provided a uniform concave nesting surface of similar dimensions and defensibility as natural nests observed in the field and were readily adopted by *P. amboinensis.*

### Experimental Design

An initial assessment of reproductive activity was conducted over a 2-day period on the contiguous reef, to establish if the breeding season had started. No reproductive activity was recorded, and no active nests were found. Behavioural interactions between males and females were recorded giving some indication that reproduction was imminent. Forty females of differing size were captured from the contiguous main reef using hand nets and a clove oil/ethanol solution. The male most closely associated with the female was also captured – forming the breeding pairs. *In situ* (underwater) the fish were sexed by visual inspection of their genital papilla and standard length (SL) was measured to the nearest millimetre using callipers through a plastic bag to prevent damage and reduce stress. Pairs were randomly chosen under the constraint that their overall size distribution should span the full range of female sizes present in the local population. The standard length of females in the population at this location ranges from 35 to 65 cm (McCormick unpublished data). To allow individual identification, all fish were tagged with a non-toxic subcutaneous fluorescent elastomer injected just beneath the epidermis [49]. Each tagged pair was haphazardly allocated and released onto an isolated experimental patch reef. Spawning activity of the fish was monitored daily within two hours of dawn, the presence of the breeding pairs (individually identifiable), and the presence of and developmental stage of eggs were recorded. Each clutch of eggs was digitally photographed *in situ* the morning of spawning. A transparent 1cm^2^ grid was placed over the clutch to facilitate measurement calibration. The area occupied by the egg mass and the density of eggs was estimated from the calibrated digital photographs using the image analysis software Optimas 6.5 (Media Cybernetics, 1999). The density was quantified by counting the number of eggs within five 10 mm^2^ areas. The number of eggs per clutch was a product of the total area occupied by the egg mass and the density of eggs. Due to the resolution of the in situ digital photographs, and the poor relationship between egg size and larval characteristics in this species [54], [55], this study did not measure egg size.

On the 5^th^ day following egg deposition at about 2–3 h before hatching of embryos, the artificial nests were collected from the patch reefs and moved to an aerated flow-through seawater system. Each nest was placed in individual aquarium maintained with aerated sea water with a unidirectional flow over the clutches. Water temperature within the aquaria was equivalent to that in the field (24–26°C). All collected nests were replaced with another nesting surface at this time.

At hatching a sample of ∼150–200 larvae was collected and placed in solution of 2.5% glutaraldehyde in seawater [56]. The larvae were fixed for two hours at room temperature (26°C), then rinsed and transferred to fresh filtered seawater and stored at 4°C. Thirty larvae from each of the 90 clutches (n = 2700 larvae) were individually photographed using a digital camera attached to a stereomicroscope. A scale bar was placed adjacent to the larvae to facilitate calibration of size measurements. The image analysis program Optimas 6.5 (Media Cybernetics, 1999) was used to measure three larvae attributes: total larval length (mm), yolk sac area (mm^2^), oil globule area (mm^2^). Each attribute was measured blind on three occasions to identify any discrepancies.

At the conclusion of the experimental period all successful breeding pairs (n = 25) were collected from the reefs using hand nets and euthanised by cold shock. All fish were measured (SL ±0.01 mm) and weighed (± 0.001 g). The sagittal otoliths and the gonads of the maternal fish were removed. The gonads were blotted dry and weighed (±0.001 g). The sagittal otoliths were prepared following Fowler [57] to determine the age of the maternal fish. Five replicate counts on the number of opaque bands were made for each fish, and any discrepancies between the measurements were identified. A linear regression model was used to determine if size and age could be considered independent variables for the female population at the study location (Table S12). The relationship between female size and age for this population is weak (Figure S1, R^2^ = 0.08), allowing us to test size and age as independent variables.

To assess the effect of both female attributes and sequential spawning on reproductive output and larval characteristics at hatching, only data collected from breeding pairs that commenced spawning within 5 days of each other, spawned at least six times over the duration of the experiment (mean = 6.4 clutches, range = 2–10 clutches) and had similar inter-brood times were used in the analysis (n = 15). This choice was made so that a valid comparison could be carried out between parents that had undertaken the same number of reproductive events over the experimental period. Examining the influence of spawning frequency (i.e. the individually variable number of clutches spawned) within the spawning period on the link between parental attributes and offspring was beyond the scope of the present study.

### Statistical analyses

#### Parental influence on reproductive output

A number of measures were calculated as indices of body condition. The residuals of a standard length by wet weight plot were used as an index of relative condition [58] for both the female and male adults. Since adults are from the same population this is generally believed to be an effective way of standardizing weight for variable body size. The further individuals differ from the general size/weight relationship, the greater or lower their body condition (i.e. they either have higher or lower than average standardized weight). Female gonad somatic index (GSI) was calculated as the ratio of ovary weight (g) to body wet weight (g), multiplied by 100. GSI gives an indication of the reproductive state at the time of sampling, and how spawning history may have impacted energy stores. All statistical analyses were conducted using Statistica version 9 (Stat Soft, Tulsa, USA).

To investigate the influence of parental characteristics on the total number of embryos produced over the six reproductive events (dependent variable) a best-subset multiple regression model using a Mallows CP selection criteria was preformed. The relationship between female size and maturity for *Pomacentrus amboinensis* at this location is weak (Figure S1), therefore we can explore the influence of both size and age on production and larval characteristics as independent variables. The parental characteristics included in the analysis were maternal size (SL), maternal age, maternal GSI, maternal condition, paternal size (SL), and paternal condition. The assumptions of multiple regression, linearity and normality, were investigated by examining bivariate scatterplot of the variables of interest and residual analysis.

#### Serial production

The influence of maternal size, age and condition on the total number of embryos produced from each successive spawn was explored using a repeated measures ANOVA. Maternal fish were grouped into three size classes (small, 47–52 mm SL, n = 5; medium, 55–60 mm SL, n = 5; and large, 63–68 mm SL, n = 5), three levels of condition (lower than average, average and better than average) and three age classes (2–3 years old n = 5, 4–5 years old n = 5, ≥6 years old n = 5) (Bonferroni adjustment was applied for repeated tests, corrected alpha  = 0.016). The assumptions of ANOVA (homogeneity of variance and normality) were examined with residual analysis. The assumption of compound symmetry was tested using Mauchley's test of sphericity [59].

#### Larval characteristics

A Friedman ANOVA with Kendall Coefficient of Concordance was used to test if there was a difference in inter-brood time among females (spawning frequency).

To explore the relationship between larval attributes (dependent variable: larval length, and energy reserves (yolk sac area and oil globule area)) and parental characteristics (independent variable) and at a particular point in the spawning sequence for *P. amboinensi*s three best subset multiple regressions were conducted. To be conservative, Bonferroni adjustment was applied for repeated tests since the analysis was conducted on three potentially co-varying dependent variables (larval length and energy reserves (2)) corrected alpha  = 0.016). The parental characteristics included in the regression models were maternal size (SL), maternal age, maternal GSI, maternal condition, paternal size (SL), and paternal condition. To standardise for prior spawning history and to reduce the chance of over estimating variability due to the variable nature of the first batch of eggs [60] the analysis was conducted on the second clutch of all spawning pairs. Multiple regression models were also conducted on the fourth and sixth clutch of all spawning pairs to test if the same adult characteristics were influential throughout the spawning season. The selection criterion was based on the model with the smallest Mallows Cp and the largest adjusted R^2^ value. The assumptions of multiple regression, linearity and normality, were investigated by examining bivariate scatterplot of the variables of interest and residual analysis.

To investigate the influence of maternal size on the three larval characteristics (i.e. larval length, yolk sac area, and oil globule area) across six successive spawning events a series of repeated measures ANOVA were conducted (Bonferroni adjusted for co-linearity between larval traits alpha = 0.016). Maternal fish were again grouped in three size classes. The assumptions of ANOVA were examined with Levene's tests, and residual analysis. The assumption of compound symmetry was tested using Mauchley's test of sphericity [59].

## Results

### Production

**Figure 1:**
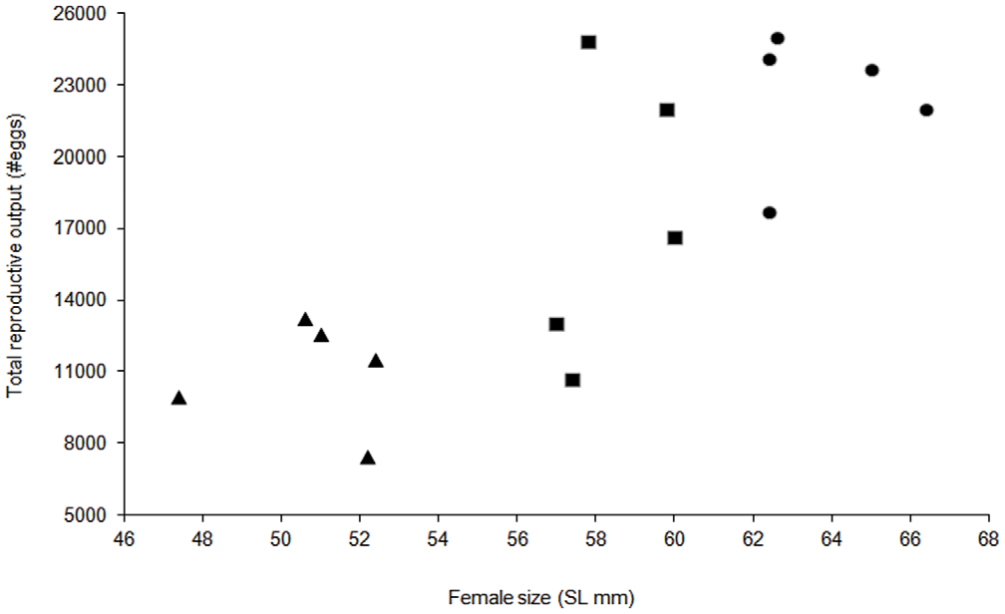
Relationship between female standard length (mm) and total reproductive output (from six clutches) over the duration of the experiment. Filled triangle represents the small females (47–52 mm; n = 5), filled square represents the medium females (55–60 mm; n = 5), and filled circle represents the large females (63–68 mm; n = 5).

Of the six parental characteristics examined, only maternal size was found to have an effect on the total number of eggs produced over the six spawning events (Table S1. adjusted R^2^ = 0.729, p = 0.0024). Total egg number increased with maternal size (Fig. 1). Large females (63–68mm SL) on average produced 51% more embryos than smaller females (47–52 mm SL) over the six spawning events.

**Figure 2:**
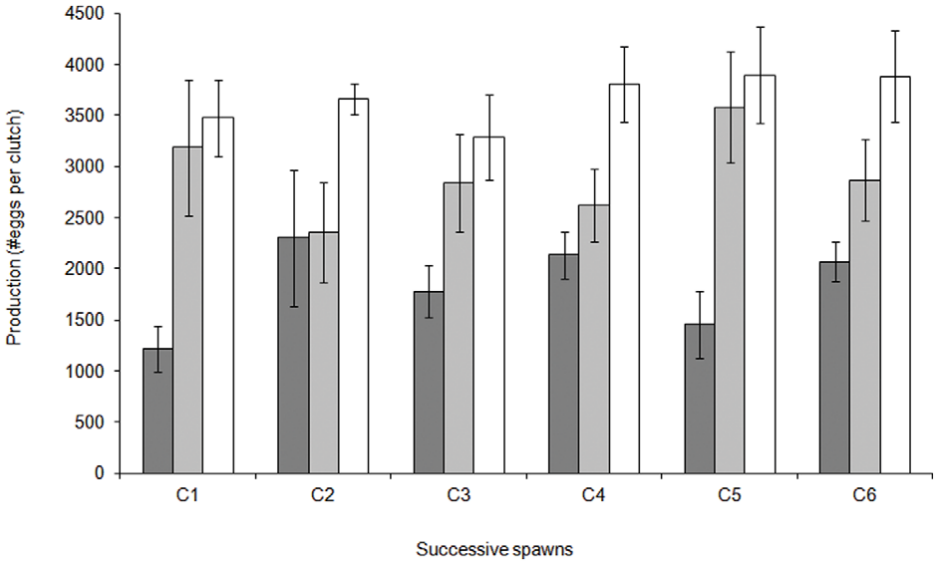
Comparison of the mean (± SE) number eggs produced per clutch between females of different size for six successive clutches. Female size had a significant influence on the number of offspring produced. C1-C6 represents six successive clutches from the same females. Dark grey bar represents the small sized females (47–52 mm; n = 5), light grey bar represents the medium sized females (55–60 mm; n = 5), and white bar represents the large sized females (63–68 mm; n = 5).

Examining the relationship between maternal characteristics and egg production for each sequential spawning event revealed an influence of maternal size but no influence of clutch number in determining the number of eggs produced (Table S2; F_10,60_  = 2.058, p = 0.006). Larger females produced significantly greater number of eggs than the smaller females across the six sequential spawning events (Fig. 2). Within each maternal size class there was no trend in the number of eggs produced across the six spawning events. There was no effect of maternal age or body condition on the number of eggs produced across the sequential spawning events (Table S2).

A Friedman ANOVA with Kendall Coefficient of Concordance was used to test if there was a difference in inter-brood time among the 15 females. No difference of inter-brood time was found (*X^2^*_(5, 14)_ = 16.0393, p = 0.3109).

### Larval characteristics

The influence of parental characteristics (maternal size, condition, GSI, and age, and paternal size and condition) on larval attributes was examined using multiple regression models. As female size was the only parental characteristic found to have influence on larval length (Table S3, adjusted R^2^ = 0.633, p = 0.0014) and larval energy reserves (Table S4, adjusted R^2^ = 0.788, p = 0.0002) throughout the spawning season (Table S3, S4, S5, S6, S7, and S8), female size is the characteristic that we have explored further in relation to larval characteristics over sequential spawns.

### Larval length

**Figure 3:**
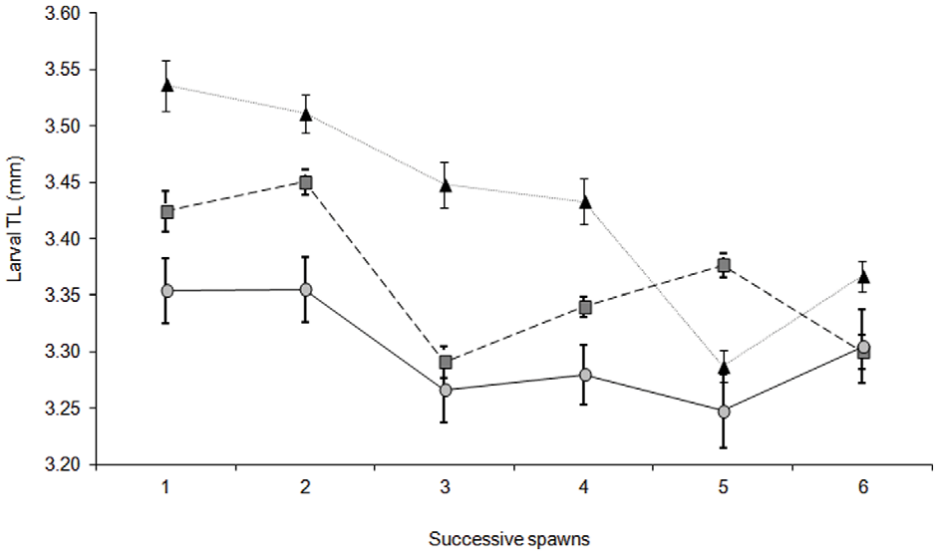
Mean (± SE) larval total length (mm) at hatching from different size females through six successive spawns. Both female size and clutch number had a significant influence on larval length (RM ANOVA; F_10, 435_ = 32.061, p = 0.0001). Filled triangle represent the small sized females (47–52 mm n = 5), filled square represents the medium sized female (55–60 mm n = 5), and filled circle represents the large sized females (63–68mm n = 5).

The total length of larvae was influenced by an interaction between maternal size and clutch number (Table S9, F_10, 435_ = 32.061, p = 0.0001). In the first clutch the smaller females produced larvae that were longer (3.54±0.02 mm, n = 5) than larvae from medium (3.42±0.02 mm) or large females (3.35±0.03 mm). This pattern was maintained across five of the six spawning events (Fig. 3). In general, there was a decline in the size of the larvae produced, irrespective of female size, as the breeding season progressed (Fig. 3). The greatest decline in larval length is seen in the small and medium sized females, declines of 4.7% and 3.6% respectively. This trend suggests that there may be a negative influence of successive spawning on larval size at hatching for the two smaller size class females.

### Energy reserves

**Figure 4:**
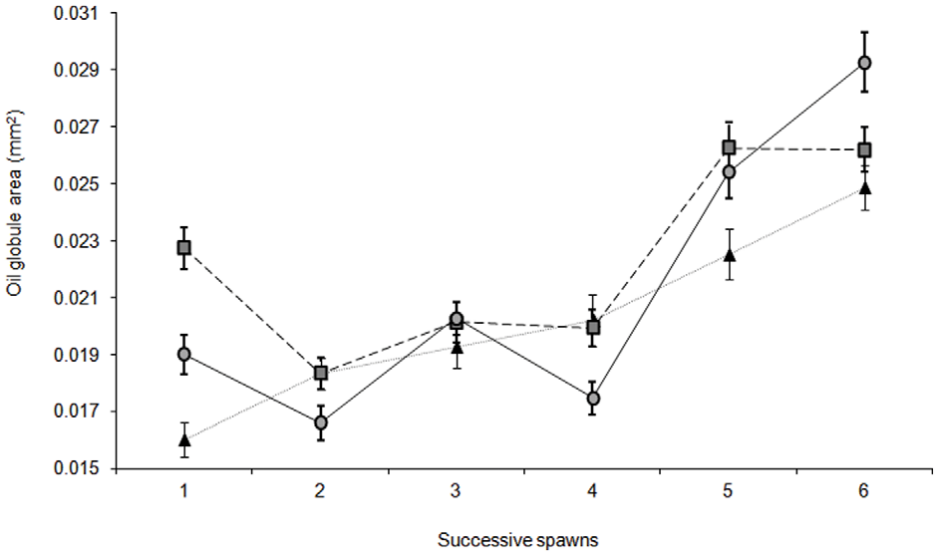
Mean (± SE) oil globule area (mm^2^) at hatching from different size females through six successive spawns. Both female size and clutch number had a significant influence on larval oil globule area (RM ANOVA; F_10, 435_ = 14.710, p = 0.0001). Filled triangle represent the small sized females (47–52 mm n = 5), filled square represents the medium sized female (55–60 mm n = 5), and filled circle represents the large sized females (63–68 mm n = 5).

**Figure 5:**
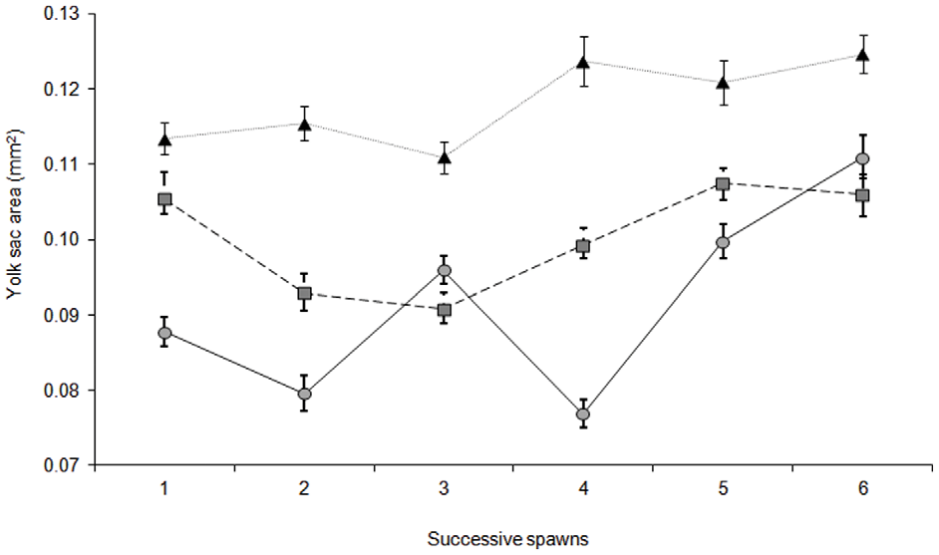
Mean (± SE) yolk sac area (mm^2^) at hatching from different size females through six successive spawns. Both female size and clutch number had a significant influence on larval yolk sac area (RM ANOVA; F_10, 435_ = 17.610, p = 0.0001). Filled triangle represent the small sized females (47–52 mm n = 5), filled square represents the medium sized female (55–60 mm n = 5), and filled circle represents the large sized females (63-68 mm n = 5).

Larval energy reserves in the form of oil globule size were significantly influenced by an interaction between female size and clutch number (Table S10. F_10, 435_  = 14.710, p = 0.0001). Fluctuations within a general trend for increasing oil globule size over the successive spawns lead to an interaction between female size and clutch (Table S10). There was a general increase in oil globule area over the successive spawns for all larvae irrespective of the maternal size (Fig. 4); however, the degree of increase differed. Oil globule area of larvae from the smallest and largest females increased by approximately 35% from clutches one to six, while the oil globule area of larvae from the medium females increased by only 13% from clutches one to six (Fig. 4.). Similarly, larval energy reserves in the form of yolk sac area were also significantly influenced by an interaction between female size and clutch number (Table S11. F_10, 435_  = 17.610, p = 0.0001). The size of the yolk sac generally increased as the spawning season progressed for larvae produced by the small females.). Greater variability in yolk sac area was seen for both the medium and large females between the spawning events (Fig. 5). Larvae produced by the smallest females had larger yolk sac area than medium or large females across all spawning events (Fig. 5). In the first spawning event small females produced larvae with a yolk sac area 29% larger than that of larvae from the large females and 8% larger than larvae from the medium sized females (Fig. 5).

## Discussion

To date the importance of between season variability in resource allocations has been demonstrated in many species (e.g. red deer, mites, moose, willow tit and gulls) with several broad ecological implications, including dramatic cohort effects on condition [61], [62], breeding success [63], [64], and survival [65]. However, at present the way organisms that serially spawn partition their energy within a reproductive season is poorly understood, particularly in tropical systems where demographic rates are often high and breeding can occur for extended periods.

In the present study we found no predictable change in egg production across the six spawning events for *P. amboinensis*. This suggests current production is unaffected by prior energy investment into reproduction, for this tropical damselfish. This was surprising, given the costs associated with reproduction, and that coral reefs are generally energy limited systems [48]. It was anticipated that the production of several large clutches (>3000 eggs per clutch) over a relatively short time period would incur an energetic cost, expressed as a decline in production over time [66].

While no influence of serial spawning on production was apparent in this study, the size of the mother influenced gross reproductive output. Larger females produced a greater number of embryos per clutch, resulting in a greater total reproductive output. The positive relationship between female size and reproductive output is well recognised and widespread in reptiles [67]–[70], amphibians [71], [72], insects [73], [74] and fishes [8], [75], [76]. Fecundity increases with body size because the amount of energy available for egg production [77], [78] and the body cavity accommodating the eggs increase with size [1], [35]. As most of these factors are interrelated to age and growth it is difficult to disentangle the effects of size and maturity for breeding populations. Although the relationship between size and age for the female *Pomacentrus amboinensis* population at the breeding site is weak, it is possible that the significant difference in egg production between smaller and larger females is due to a covariance of size and maturity. We would like to acknowledge that it is possible that we are comparing first time spawners to those that have spawned in previous years, a variable we cannot determine or control for when sampling from a wild population. Fish life history models commonly contain direct correlations between fecundity and a female's age or size [1], [79]. Yet there is a pronounced absence in fish life history theory of consideration for variation in offspring quality or viability with change in parent size or reproductive effort [7]. Production is only one element of the complex of factors that influence the number of offspring that survive from a spawning. Exploring only the quantity of eggs a female produces provides limited power to predict individual fitness.

Investigating characteristics of the offspring at hatching provided us with greater information on reproductive allocation in this iteroparous species, and allowed us to hypothesize benefits of spawning multiple times within a reproductive season. We found that smaller females produced both the longest larvae and larvae with greater energy reserves. This result is curious and contrary to many studies that have found a positive relationship between female size and egg size [80]–[82]. One possible explanation for the pattern is that there is a morphological restriction to the number of offspring a small female can produce and therefore excess reserves are allocated to offspring quality. A study conducted on the bicolor damsel-fish (*Stegastes partitus*) found that when females were supplemented with additional resources there was no change in the number of offspring produced but the quality of the offspring changed [47]. The author suggests that production is mediated by population demographics which change over longer time scales, whereas offspring quality may change in response to short term variation in the environment. A short term change in the condition of *P. amboinensis* offspring has been observed in response to an increase in natural food availability [26].

In our study the characteristics of the offspring changed significantly over the breeding season. Over the six spawns there was a general decline in larval length, which was most apparent in larvae from the small females. Little research has investigated the traits of the resulting larvae from successively produced clutches. Declines in the size of eggs produced successively have been detected in a few species of turtles: *Kinosternon subrubrum*, *Sternotherus odoratus*, and *Pseudemys floridana* [69] and fishes: *Gadus morhua* [83], and *Rhinogobius sp*. [84]. Whether or not a decline in the size of the eggs produced has a fitness consequence for the resulting offspring is yet to be fully explored. Egg size may not always be a good proxy for egg quality and may not accurately reflect maternal investment in reproduction [85]. Larvae of shorter length but with larger reserves could occupy the same size egg as larvae of longer length but with fewer reserves.

Exploring only one larval characteristic at hatching can lead to invalid conclusions of the workings of this complex system. For example, in this study we could have assumed an associated cost to the quality of offspring when produced successively, expressed as a decline in larval length. However, both the size of the oil globule within the yolk and the size of the yolk-sac increased over successive reproductive events. Lipids contained within the oil globule are believed to be a principal source of energy after hatching and important for post-hatching longevity [86].

In this study we have demonstrated that in a serially spawning fish the characteristics of offspring vary over relatively short time periods. Initially offspring were longer in length and had fewer energy reserves, but as the season progressed offspring were characterised by shorter length but with larger energy reserves. One explanation for the variation in offspring characteristic observed in this study is that by producing offspring that vary the parents ensure that some offspring will survive no matter what environmental conditions are encountered [46]. *Pomacentrus amboinensis* offspring disperse away from the parent environment. Therefore, the parents cannot directly predict the environmental conditions where development will take place. Consequently, we may not expect to find a strong link between the offspring's provisioning and parents' environment at the time of gametogenesis. The production of variable offspring may be under parental control, it may not however be a direct response to the environmental conditions encountered by the parents during the reproductive process.

Alternatively, the change in offspring characteristics may reflect selection for larger reserves later in the season. Offspring spawned later in the season will recruit to a reef system where several other cohorts have already established residence. The more eggs the mothers lay, the greater the likelihood that the offspring will recruit to a population with higher density. Females that produce better provisioned offspring later in the season should have enhanced reproductive success, by making their offspring more competitive. Apportioning more energy to each larval offspring should enhance maternal fitness [85] as endogenous reserves influence initial larval growth [87], and fast-growing, early-metamorphosing damselfish show the highest larval survivorship [88], [89]. Therefore, selection for larger reserves later in the season may not only enhance larval survivorship but may also create equality between all offspring produced over the season. By allocating resource differently over the reproductive season it is possible that the parents are mediating competition for resources. This type of resource allocation has been seen in several species where there is high competition for resources between offspring [43], [90].

Studies investigating the importance of larval characteristics at the time of recruitment in *P.amboinensis* provide evidence that mortality is a selective process and certain characteristics increase survival probability. Hoey and McCormick [91] found that larvae of lower total lipid content and low pre-settlement growth rates were selectively preyed upon. They also found that total lipid content was negatively related to standard length, suggesting that smaller recruits had a tendency to have a higher proportion of lipid than larger recruits [91]. Similarly, Meekan et al. [92] found that larger, faster growing *P.amboinensis* larvae suffered high mortality rates at settlement. It appears that body condition, in the form of available or excess energy, is important at settlement and may determine which individuals survive this period of high selection [91], [92].Whether or not the phenotypic characteristics offspring have at hatching are maintained over the entire larval phase remains in doubt, as it is very difficult to determine [93].

The findings of this study highlight that examining a single reproductive event only provides a snapshot of the investment into reproduction for multiple spawning species. Caution is needed when extrapolating total successful reproductive output from one clutch. Our results also highlight the variability in early life history characteristics among sequential reproductive events. This stresses the importance of assessing the quality of larvae in addition to quantity when investigating the fitness consequences to successively spawned offspring. If fitness consequences of iteroparity are to be accurately evaluated, life history models should be appropriately revised to include information on the influence of successive spawns on production, larval quality and viability [94].

In summary, evidence suggests that small females produced larvae of higher quality. All females regardless of size show flexible resource allocation, producing larvae that vary in quality over the successively produced clutches. Variation in offspring characteristics may not only enhance chances of offspring survival in the patchy pelagic environment into which they are liberated, but may also mediate density dependent resource competition between offspring. These finding may have implication for all animals that reproduce multiple times within a breeding season such as insects, reptiles and amphibians.

## Supporting Information

Figure S1 **Relationship between size and age (y = 2.3215× = 41.782, adjusted R2 = 0.083) for the female population of Pomacentrus amboinensis in the Blue Lagoon at Lizard Island, QLD Australia.** The filled gray diamonds represent the female population at the study site and the filled black squares represent the females used in the study.
(DOCX)

Table S1 **Relationship between total reproductive output with female standard length, age, GSI and body condition (BC), and male standard length and body condition (BC) at the conclusion of the six week experiment.**
(DOCX)

Table S2 **Influence of successive spawns (clutch) of females of different (a) standard length (size), (b) age and (c) condition on the total number of embryos produced.** Results are from a repeated measure analysis of variance involving repeated sampling of egg clutches from six successive spawns of individually identified females of differing sizes (corrected alpha  = 0.016).
(DOCX)

Table S3 **Relationship between larval length (dependent variable) from clutch 2 and female standard length, age, GSI and body condition (BC), and male standard length and body condition (BC).** Using a best sub set regression model.
(DOCX)

Table S4 **Relationship between larval energy reserves (dependent variable) from clutch 2 and female standard length, age, GSI and body condition (BC), and male standard length and body condition (BC).** Using a best sub set regression model.
(DOCX)

Table S5 **Relationship between larval length (dependent variable) from clutch 4 and female standard length, age, GSI and body condition (BC), and male standard length and body condition (BC).** Using a best sub set regression model.
(DOCX)

Table S6 **Relationship between larval energy reserves (dependent variable) from clutch 4 and female standard length, age, GSI and body condition (BC), and male standard length and body condition (BC).** Using a best sub set regression model.
(DOCX)

Table S7 **Relationship between larval length (dependent variable) from clutch 6 and female standard length, age, GSI and body condition (BC), and male standard length and body condition (BC).** Using a best sub set regression model.
(DOCX)

Table S8 **Relationship between larval energy reserves (dependent variable) from clutch 6 and female standard length, age, GSI and body condition (BC), and male standard length and body condition (BC).** Using a best sub set regression model.
(DOCX)

Table S9 **Influence of successive spawns of females of different standard length (size), on the length of the larvae produced.** Results are from a repeated measures analysis of variance involving repeated sampling of larvae from six successive spawns of individually identified females of differing size.
(DOCX)

Table S10 **Influence of successive spawns of females of different standard length (size) on the oil globule area of the larvae produced.** Results are from a repeated measures analysis of variance involving repeated sampling of larvae from six successive spawns of individually identified females of differing size.
(DOCX)

Table S11 **Influence of successive spawns of females of different standard length (size) on the yolk sac area of the larvae produced.** Results are from a repeated measures analysis of variance involving repeated sampling of larvae from six successive spawns of individually identified females of differing size.
(DOCX)

Table S12 **Results from a linear regression analysis conducted on the relationship between size and age for the female population of P.amboinensis at the breeding site, Blue Lagoon Lizard Island, Australia (McCormick, unpublished data).**
(DOCX)
