# Supplementary material for: Not All Offspring Are Created Equal: Variation in Larval Characteristics in a Serially Spawning Damselfish
Source: PLoS One. 2012 Nov 14;7(11):e48525. doi: 10.1371/journal.pone.0048525 (PMC3498294; doi:10.1371/journal.pone.0048525)
Supplement: Table S1 — Relationship between total reproductive output with female standard length, age, GSI and body condition (BC), and male standard length and body condition (BC) at the conclusion of the six week experiment. (DOCX) [file pone.0048525.s002.docx]

Table S1

| Parental attribute | Beta | t(8) | p-level | Adjusted R^2^ |
| --- | --- | --- | --- | --- |
| Female size | 1.7897 | 4.3512 | **0.0024** | **0.729** |
| Female age | -0.7477 | -2.4231 | 0.0516 |  |
| Female BC | 0.0605 | 0.3346 | 0.7464 |  |
| Female GSI | 0.7093 | 2.3924 | 0.0536 |  |
| Male length | 0.0399 | 0.2007 | 0.8459 |  |
| Male BC | 0.3116 | 1.9330 | 0.0893 |  |
