# Supplementary material for: Not All Offspring Are Created Equal: Variation in Larval Characteristics in a Serially Spawning Damselfish
Source: PLoS One. 2012 Nov 14;7(11):e48525. doi: 10.1371/journal.pone.0048525 (PMC3498294; doi:10.1371/journal.pone.0048525)
Supplement: Table S2 — Influence of successive spawns (clutch) of females of different (a) standard length (size), (b) age and (c) condition on the total number of embryos produced. Results are from a repeated measure analysis of variance involving repeated sampling of egg clutches from six successive spawns of individually identified females of differing sizes (corrected alpha = 0.016). (DOCX) [file pone.0048525.s003.docx]

Table S2

| Source | df | MS | F | p |
| --- | --- | --- | --- | --- |
| a. Female size | 2 | 27936977 | 10.2421 | **0.0056** |
| Error | 12 | 2727651 |  |  |
| Clutch | 5 | 549141 | 1.1342 | 0.5452 |
| Clutch*Female size | 10 | 996564 | 2.0583 | 0.0475 |
| Error | 60 | 484157 |  |  |
| b. Female age | 3 | 14155354 | 3.2654 | 0.0573 |
| Error | 10 | 4334907 |  |  |
| Clutch | 5 | 728424 | 1.3015 | 0.4741 |
| Clutch*Female age | 15 | 596227 | 1.0653 | 0.8474 |
| Error | 50 | 559661 |  |  |
| c. Female condition | 2 | 1274428 | 0.17771 | 0.8394 |
| Error | 12 | 7171409 |  |  |
| Clutch | 5 | 583623 | 1.03719 | 0.4043 |
| Clutch*Female condition | 10 | 525343 | 0.93362 | 0.5095 |
| Error | 60 | 562694 |  |  |
