# Supplementary material for: Not All Offspring Are Created Equal: Variation in Larval Characteristics in a Serially Spawning Damselfish
Source: PLoS One. 2012 Nov 14;7(11):e48525. doi: 10.1371/journal.pone.0048525 (PMC3498294; doi:10.1371/journal.pone.0048525)
Supplement: Table S3 — Relationship between larval length (dependent variable) from clutch 2 and female standard length, age, GSI and body condition (BC), and male standard length and body condition (BC). Using a best sub set regression model. (DOCX) [file pone.0048525.s004.docx]

Table S3

| Parental attribute | Beta | t(11) | p-level | Adjusted R^2^ |
| --- | --- | --- | --- | --- |
| Female size | -0.679 | -4.215 | **0.001** | **0.633** |
| Female age | -0.747 | -2.423 | 0.052 |  |
| Female BC | -0.432 | -2.509 | 0.029 |  |
| Female GSI | 0.709 | 2.392 | 0.054 |  |
| Male length | 0.039 | 0.200 | 0.846 |  |
| Male BC | 0.204 | 1.189 | 0.259 |  |
