# Supplementary material for: Not All Offspring Are Created Equal: Variation in Larval Characteristics in a Serially Spawning Damselfish
Source: PLoS One. 2012 Nov 14;7(11):e48525. doi: 10.1371/journal.pone.0048525 (PMC3498294; doi:10.1371/journal.pone.0048525)
Supplement: Table S4 — Relationship between larval energy reserves (dependent variable) from clutch 2 and female standard length, age, GSI and body condition (BC), and male standard length and body condition (BC). Using a best sub set regression model. (DOCX) [file pone.0048525.s005.docx]

Table S4

| Parental attribute | Beta | t(11) | p-level | Adjusted R^2^ |
| --- | --- | --- | --- | --- |
| Female size | -0.797 | -2.415 | **0.0002** | **0.788** |
| Female age | -0.276 | -1.307 | 0.220 |  |
| Female BC | -0.432 | -2.509 | 0.029 |  |
| Female GSI | -0.272 | -1.093 | 0.300 |  |
| Male length | 0.039 | 0.201 | 0.846 |  |
| Male BC | 0.357 | -2.041 | 0.068 |  |
