# Supplementary material for: Not All Offspring Are Created Equal: Variation in Larval Characteristics in a Serially Spawning Damselfish
Source: PLoS One. 2012 Nov 14;7(11):e48525. doi: 10.1371/journal.pone.0048525 (PMC3498294; doi:10.1371/journal.pone.0048525)
Supplement: Table S5 — Relationship between larval length (dependent variable) from clutch 4 and female standard length, age, GSI and body condition (BC), and male standard length and body condition (BC). Using a best sub set regression model. (DOCX) [file pone.0048525.s006.docx]

Table S5

| Parental attribute | Beta | t(11) | p-level | Adjusted R^2^ |
| --- | --- | --- | --- | --- |
| Female size | -0.625 | -4.362 | **0.005** | **0.521** |
| Female age | -0.777 | -2.568 | 0.051 |  |
| Female BC | -0.567 | -2.802 | 0.069 |  |
| Female GSI | 0.060 | 0.334 | 0.073 |  |
| Male length | 0.035 | 0.300 | 0.765 |  |
| Male BC | 0.209 | 1.198 | 0.361 |  |
