# Supplementary material for: Not All Offspring Are Created Equal: Variation in Larval Characteristics in a Serially Spawning Damselfish
Source: PLoS One. 2012 Nov 14;7(11):e48525. doi: 10.1371/journal.pone.0048525 (PMC3498294; doi:10.1371/journal.pone.0048525)
Supplement: Table S6 — Relationship between larval energy reserves (dependent variable) from clutch 4 and female standard length, age, GSI and body condition (BC), and male standard length and body condition (BC). Using a best sub set regression model. (DOCX) [file pone.0048525.s007.docx]

Table S6

| Parental attribute | Beta | t(11) | p-level | Adjusted R^2^ |
| --- | --- | --- | --- | --- |
| Female size | -0.803 | -3.021 | **0.002** | **0.623** |
| Female age | -0.302 | -1.650 | 0.138 |  |
| Female BC | -0.510 | -2.511 | 0.089 |  |
| Female GSI | -0.252 | -1.201 | 0.209 |  |
| Male length | 0.052 | 0.304 | 0.789 |  |
| Male BC | 0.489 | -3.002 | 0.568 |  |
