# Supplementary material for: Not All Offspring Are Created Equal: Variation in Larval Characteristics in a Serially Spawning Damselfish
Source: PLoS One. 2012 Nov 14;7(11):e48525. doi: 10.1371/journal.pone.0048525 (PMC3498294; doi:10.1371/journal.pone.0048525)
Supplement: Table S7 — Relationship between larval length (dependent variable) from clutch 6 and female standard length, age, GSI and body condition (BC), and male standard length and body condition (BC). Using a best sub set regression model. (DOCX) [file pone.0048525.s008.docx]

Table S7

| Parental attribute | Beta | t(11) | p-level | Adjusted R^2^ |
| --- | --- | --- | --- | --- |
| Female size | -0.792 | -5.415 | **0.014** | **0.508** |
| Female age | -0.695 | -2.649 | 0.056 |  |
| Female BC | -0.532 | -2.709 | 0.078 |  |
| Female GSI | 0.793 | 2.439 | 0.086 |  |
| Male length | 0.0608 | 0.108 | 0.798 |  |
| Male BC | 0.402 | 1.207 | 0.324 |  |
