# Supplementary material for: Not All Offspring Are Created Equal: Variation in Larval Characteristics in a Serially Spawning Damselfish
Source: PLoS One. 2012 Nov 14;7(11):e48525. doi: 10.1371/journal.pone.0048525 (PMC3498294; doi:10.1371/journal.pone.0048525)
Supplement: Table S8 — Relationship between larval energy reserves (dependent variable) from clutch 6 and female standard length, age, GSI and body condition (BC), and male standard length and body condition (BC). Using a best sub set regression model. (DOCX) [file pone.0048525.s009.docx]

Table S8

| Parental attribute | Beta | t(11) | p-level | Adjusted R^2^ |
| --- | --- | --- | --- | --- |
| Female size | -0.684 | -2.089 | **0.018** | **0.597** |
| Female age | -0.287 | -1.181 | 0.302 |  |
| Female BC | -0.509 | -2.432 | 0.092 |  |
| Female GSI | -0.399 | -1.207 | 0.203 |  |
| Male length | 0.027 | 0.109 | 0.984 |  |
| Male BC | 0.573 | -2.320 | 0.085 |  |
