# Supplementary material for: Not All Offspring Are Created Equal: Variation in Larval Characteristics in a Serially Spawning Damselfish
Source: PLoS One. 2012 Nov 14;7(11):e48525. doi: 10.1371/journal.pone.0048525 (PMC3498294; doi:10.1371/journal.pone.0048525)
Supplement: Table S9 — Influence of successive spawns of females of different standard length (size), on the length of the larvae produced. Results are from a repeated measures analysis of variance involving repeated sampling of larvae from six successive spawns of individually identified females of differing size. (DOCX) [file pone.0048525.s010.docx]

Table S9

| Source | df | MS | F | p |
| --- | --- | --- | --- | --- |
| Female size | 2 | 0.63780054 | 181.783 | 0.0001 |
| Error | 87 | 0.00350858 |  |  |
| Clutch | 5 | 0.28763043 | 102.503 | 0.0001 |
| Clutch*Female size | 10 | 0.08996524 | 32.061 | 0.0001 |
| Error | 435 | 0.00280605 |  |  |
