# Supplementary material for: Not All Offspring Are Created Equal: Variation in Larval Characteristics in a Serially Spawning Damselfish
Source: PLoS One. 2012 Nov 14;7(11):e48525. doi: 10.1371/journal.pone.0048525 (PMC3498294; doi:10.1371/journal.pone.0048525)
Supplement: Table S12 — Results from a linear regression analysis conducted on the relationship between size and age for the female population of P.amboinensis at the breeding site, Blue Lagoon Lizard Island, Australia (McCormick, unpublished data). (DOCX) [file pone.0048525.s013.docx]

Table S12

| Multiple R | Multiple R^2^ | Adjusted R^2^ | SS Model | df Model | MS Model | SS Residual | df Residual | MS Residual | F | P |
| --- | --- | --- | --- | --- | --- | --- | --- | --- | --- | --- |
| 0.313 | 0.098 | 0.083 | 4.856 | 1 | 4.856 | 44.681 | 63 | 0.709 | 6.847 | 0.011 |
